# Compartment Syndrome of the Hand Secondary to Intravenous Extravasation

**Published:** 2018-09-24

**Authors:** Cara M. Barber, Matthew P. Fahrenkopf, Nicholas S. Adams, John P. Kelpin, Johanna R. Krebiehl

**Affiliations:** ^a^Michigan State University College of Human Medicine, Grand Rapids; ^b^Spectrum Health/Michigan State University Plastic Surgery Residency, Grand Rapids; ^c^Plastic Surgery Associates, Grand Rapids, Mich

**Keywords:** compartment syndrome, hand, fasciotomy, trauma, extravasation

## DESCRIPTION

**Figure 1:**
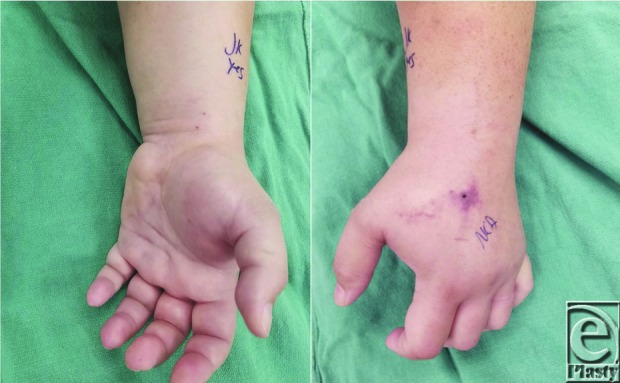
Extravasation injury to the left hand. Note loss of skin wrinkles on the dorsum of the hand.

An intubated 47-year-old woman postoperative day 1 following spinal surgery was noted to have a large volume of presumed crystalloid intravenous solution extravasation through a peripheral intravenous tube in her left hand. The infiltration went unnoticed for hours as the patient was sedated (Fig 1). After the patient was extubated, she complained of progressive sensorimotor deficits. Compartment syndrome of the hand was suspected, and hand fasciotomies were performed.

## QUESTIONS

1. What is compartment syndrome and what are the causes?
2. How does intravenous extravasation play a role in compartment syndrome?
3. How is compartment syndrome diagnosed?
4. What are the treatment options?

## DISCUSSION

Compartment syndrome of the hand is relatively uncommon finding.^1^ Compartment syndrome occurs when there is an increase in compartmental contents that leads to increased interstitial fluid pressure, thereby leading to capillary bed collapse, decreased perfusion, and eventual cell death.^1^^,^^2^ This syndrome has a cyclic pattern that compounds the pressure and edema found through subsequent cell deaths, which allow more fluid to accumulate. Compartment syndrome has many etiologies such as trauma, burns, muscle overuse, infection, bites, bleeding, and extravasation, to name a few.^1^^-^4 During compartment syndrome, nerve axonal transport slows down with a compartment pressure of 30 mm Hg or more, leading to nerve function loss. Muscles are also greatly affected by compartment syndrome and can be irreversibly damaged if left untreated within the first 6 to 8 hours.^1^^,^^2^

The relationship between intravenous extravasation and compartment syndrome is a particularly rare finding.^3^^-^5 There have been some reports that show the linkage between intravenous extravasation and subsequent compartment syndrome, but overall data toward this subject are very limited. In a recent systematic review of 51 infiltration-related compartment syndrome cases, 20 occurred in the hand.^3^ The overwhelming risk factor for infiltration-related compartment syndrome was age, with 40% of cases occurring in the pediatric population.^3^ Additional risk factors included pressurized infusion systems and barriers to doctor-patient communication, seen in patients with altered mental status or mechanical ventilation.^3^^,^^4^ They also found that physiologic intravenous fluid solutions or radiographic contrasts were the causative agent in more than 70% of cases.^3^

The diagnosis of compartment syndrome is primarily made through physical examination. The 5 P's, pain, pallor, paresthesias, paralysis, and pulselessness, are key findings in compartment syndrome. However, with the variability of findings all of these concurrently, pain with passive stretch and disproportionate pain are the most reliable findings.^2^^,^^3^ The definitive diagnosis can be confirmed by intracompartmental pressure measurement. Compartment pressures greater than 30 mm Hg or those that are within 30 mm Hg of the diastolic pressure provide an indication for fasciotomy in the proper clinical setting. However, available devices are imprecise, invasive, and highly user-dependent.^2^^,^^3^ Therefore, most compartment syndromes are diagnosed clinically. It is even more difficult to diagnose compartment syndrome early in the case of an anesthetized or obtunded patient who is incapable of expressing pain. Frequent examination of the extremities is crucial to make a timely diagnosis and minimize the risk of permanent injury. In the presence of other risk factors (long surgery, prone position, large fluid resuscitation, unknown amount of infiltrate, prolonged limb compression), physicians should have a low threshold for measuring compartment pressures or performing fasciotomies in this select group.^4^^-^6

**Figure 2:**
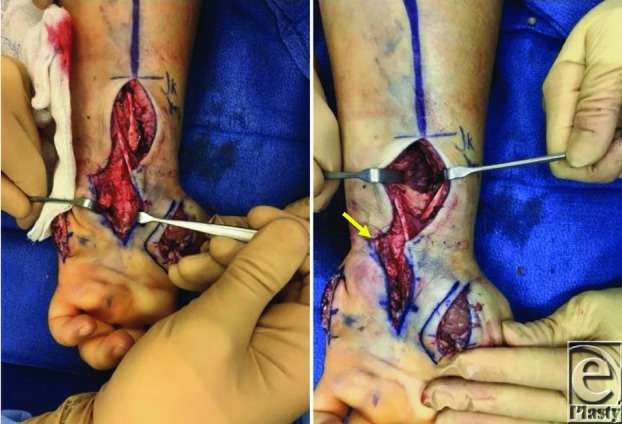
Carpal tunnel release (left, right) and distal forearm fasciotomy (right). Note the ulnar extension of the carpal tunnel incision across the wrist (arrow).

**Figure 3:**
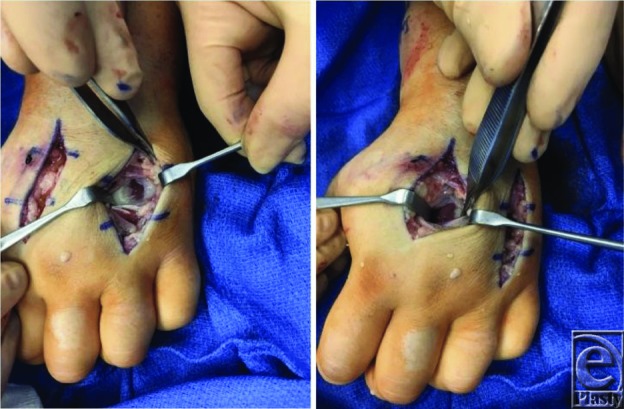
Dorsal incisions showing release of interosseous fascia.

**Figure 4:**
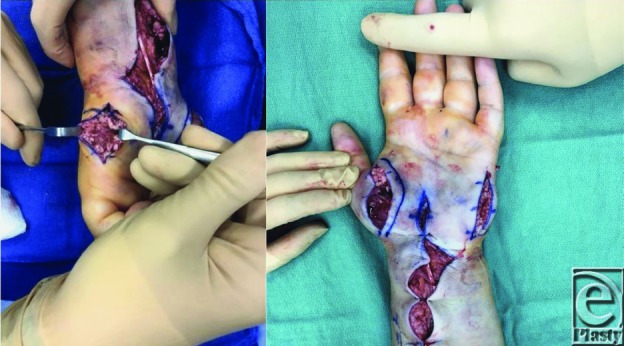
Release of thenar compartment (left) and release of adductor and hypothenar compartments (right).

Once a clinical diagnosis has been established, fasciotomies should be performed. Fasciotomies decompress the pressure within the compartments, allowing for subsequent edema without neurovascular compromise.^1^^-^4 Guidelines for hand fasciotomies advocate the release of all 10 intrinsic hand compartments (thenar, hypothenar, adductor, 4 dorsal interossei, and 3 volar interossei) in addition to the carpal tunnel (Figs 2-4).^1^^-^3^,^^7^ The interossei are released using the 2 dorsal incisions centered over the second and fourth metacarpals (Fig 3). Separate incisions are made over the adductor, hypothenar, and thenar compartments to permit their respective decompressions (Figs 2 and 4). The carpal tunnel incision is designed along the radial border of the ring finger. When extending the carpal tunnel incision across the wrist, it is carried ulnarly to avoid and protect the palmar cutaneous branch of the median nerve (Fig 2). A recent cadaver study did note that there was substantial variability in fascial distribution and that dense fascia did not always encase all of the intrinsic hand muscles.^8^ They postulated that the unyielding overlying skin may serve as an additional constricting layer along with the intrinsic muscular fascia and contribute to raised intracompartmental pressures.^8^

In the case of the aforementioned patient, crystalloid intravenous extravasation, of an unknown amount, was suspected as the cause of her compartment syndrome. This went unnoticed for several hours as the patient was intubated at the time. After extubation, the patient complained of worsening hand pain and paresthesias, so the hand service was consulted. She was initially treated with elevation and experienced improvement in symptoms. However, serial examinations demonstrated worsening 2-point discrimination in her thumb and ring fingers and loss of sensation in the index and long fingers. She was clinically diagnosed with compartment syndrome and treated with hand fasciotomies. She went on to recover all sensation in her hand without functional disability.

Remember, the majority of intravenous extravasation injuries are benign and may be treated with conservative measures (elevation, time). Serial examinations over the first 24 to 48 hours are paramount to detect any change in clinical status. In patients who fail to respond to these therapies, a low threshold must be kept for operative intervention. Specifically, those individuals who display stagnant or worsening physical examination findings (neurologic changes, vascular compromise, increasing pain), in the presence of other risk factors (long surgery, prone position, large fluid resuscitation, unknown amount of infiltrate, sedation) should have compartment pressures measured and/or fasciotomies performed. The morbidity of fasciotomies remains relatively low, while the consequences of misdiagnosing compartment syndrome can be significant and lead to lifelong patient disabilities.^1^^,^^4^^,^^6^
